# Severe Invasive Pneumococcal Infection With Multiple Abscesses Caused by a Less Virulent Serotype 1 Pneumococcus: A Case Report

**DOI:** 10.7759/cureus.79398

**Published:** 2025-02-21

**Authors:** Takae Okuno, Megumi Hamaguchi, Amano Yoshihiro, Mika Nakao, Takeshi Isobe

**Affiliations:** 1 Department of Internal Medicine, Division of Medical Oncology and Respiratory Medicine, Shimane University Faculty of Medicine, izumo, JPN; 2 Department of Internal Medicine, Division of Medical Oncology and Respiratory Medicine, Shimane University Faculty of Medicine, Izumo, JPN

**Keywords:** case report, empyema, invasive pneumococcal disease, non-vaccine serotype, serotype replacement

## Abstract

Invasive pneumococcal disease (IPD) has a high fatality rate; however, its severity varies by pneumococcal serotype. Serotype 1 *pneumococcus *is often associated with empyema but typically has a low fatality rate, and IPD is rarely reported in such cases. We report the case of a 68-year-old man who developed IPD with systemic abscesses, including empyema, purulent pericardial effusion, and an intramuscular abscess in the right thigh, along with sepsis caused by serotype 1 *pneumococcus*. He remained hospitalized for 10 months. This level of disease severity may have been preventable if the patient had obtained serotype 1 immunity through the pneumococcal conjugate vaccine (PCV13) and the 23-valent pneumococcal polysaccharide vaccine (PPSV23). This case highlights the critical importance of pneumococcal vaccination for older adults and immunocompromised individuals.

## Introduction

According to the 2019 Japanese Vital Statistics, pneumonia is the fifth leading cause of death in Japan, with more than 97% of pneumonia-related deaths occurring in individuals aged 65 years and older [1]. This makes pneumonia a major health concern for the elderly population. *Streptococcus pneumoniae*, the causative agent in one-quarter of all pneumonia cases [2], can also lead to invasive pneumococcal disease (IPD). IPD is defined as a serious infectious disease, such as meningitis or sepsis, resulting from pneumococcal infection in normally sterile areas, including the cerebrospinal fluid and blood. This condition requires particular attention in children, the elderly, and immunocompromised individuals due to its severity and high risk of complications. Because IPD can be fatal, prevention and early therapeutic intervention are critical. However, the severity of IPD depends on the serotype; *Streptococcus pneumoniae* is classified into more than 90 serotypes, based on the antigenicity of the capsular membrane [3]. The pneumococcal vaccine has been shown to reduce the incidence of IPD [4], pneumonia-related mortality in the elderly, and pneumococcal community-acquired pneumonia [5,6], underscoring the importance of vaccination for infection prevention. Here, we present the case of a 65-year-old man who was not vaccinated against *Streptococcus pneumoniae* and subsequently developed severe IPD due to an attenuated serotype 1 pneumococcal infection.

## Case presentation

A 68-year-old man presented to a local hospital with complaints of anorexia and general malaise that had persisted for one week. His comorbid conditions included hypertension, diabetes mellitus, fatty liver, dyslipidemia, and hyperuricemia. He was obese, with a height of 179 cm, weight of 97.7 kg, and a body mass index (BMI) of 30.5. His medications included amlodipine 5 mg, candesartan 8 mg, and febuxostat 20 mg. His diabetes was borderline, so he was managed with dietary measures alone. There was no history of smoking or pneumococcal vaccination, but it was noted that a few days before the onset of illness, he had spent time with his elementary school-aged granddaughter, who had a rhinovirus infection. On examination at the local hospital, he was found to have hypotension, hypoxemia, and tachycardia. A CT scan revealed pneumonia, loculated pleural effusion, and pericardial effusion. He was diagnosed with a severe infection and was transferred to our hospital via emergency transport on the same day. On admission to our hospital, his physical examination showed no change in level of consciousness (Glasgow Coma Scale: 15). His temperature was 35.6°C, blood pressure 88/44 mmHg, heart rate 140 beats per minute, respiratory rate 35 breaths per minute, and SpO₂ 93% on 12 L/min of oxygen. There was no neck stiffness and no abnormal heart rhythm or murmur. However, coarse crackles were heard in both lungs. The abdomen was flat and non-tender, and there were no skin abnormalities, but pain in the right thigh and edema in the lower extremities were noted. Blood tests revealed neutrophil-predominant leukocytosis, elevated C-reactive protein (CRP), and positive procalcitonin (PCT) levels, indicating infection. The detection of hypoproteinemia and hypoalbuminemia suggested metabolic changes due to inflammation. Blood gas analysis showed increased lactate levels and acidosis, indicating organ damage due to septic shock. Liver and kidney dysfunction were observed. An increase in creatine kinase (CK) levels suggested muscle destruction due to infection or abscess formation (Table 1).

**Table 1:** Laboratory findings: blood tests, blood gas analysis, and pleural fluid examination WBC: white blood cell, Hb: hemoglobin, PLT: platelets, Neut: neutrophils, TP: total protein; T-bil: bilirubin, AST: aspartate aminotransferase, ALT: alanine aminotransferase, LDH: lactate dehydrogenase, CK: creatinine kinase, BUN: blood urea nitrogen, Crea: creatinine, eGFR: estimated glomerular filtration rate, Na: sodium, K: potassium, Cl: chloride, CRP: C-reactive protein, Gul: glucose; HbA1c: hemoglobin A1c, PCT: procalcitonin, PCO_2_: partial pressure of carbon dioxide, PO_2_, partial pressure of oxygen, HCO_3_: peroxide, Lac: lactoferrin.

Laboratory	On admission	Reference range
WBC	28.02 ×10^3^/μL	3.30-8.60
Hb	15.8 ×g/dL	13.7-16.8
PLT	218 ×10^3^/μL	158-348
Neut	23.26 ×10^3^/μL	-
TP	4.6 g/dL	6.6-8.1
T-Bil	0.7 mg/dL	0.4-1.5
AST	79 U/L	13-30
ALT	33 U/L	10-42
LDH	498 U/L	498
CK	373 U/L	59-248
BUN	151.9 mg/dL	8.0-20.0
Crea	4.79 mg/dL	0.65-1.07
eGFR	10.5 mL/min/BSA	-
Na	125 mmol/L	138-145
K	5.3 mmol/L	3.6-4.8
Cl	89 mmol/L	101-108
CRP	31.99 mg/dL	<0.14
Gul	155 mg/dL	73-109
HbA1c	6.9%	4.9-6.0
PCT	131.08 ng/mL	<0.05
Blood gas analysis（oxygen 12 L/min）		
PH	7.238	7.35-7.45
PCO_2_	39.2 mmHg	35.0-48.0
PO_2_	152.0 mmHg	83.0-108.0
HCO_3_	16.1 mmol/L	21.0-28.0
Lac	40.0 mg/dL	4.5-13.5
Pleural effusion		
PH	6.203	-
Glu	6 mg/dL	-
PT	4.5 g/dL	-

**Figure 1:**
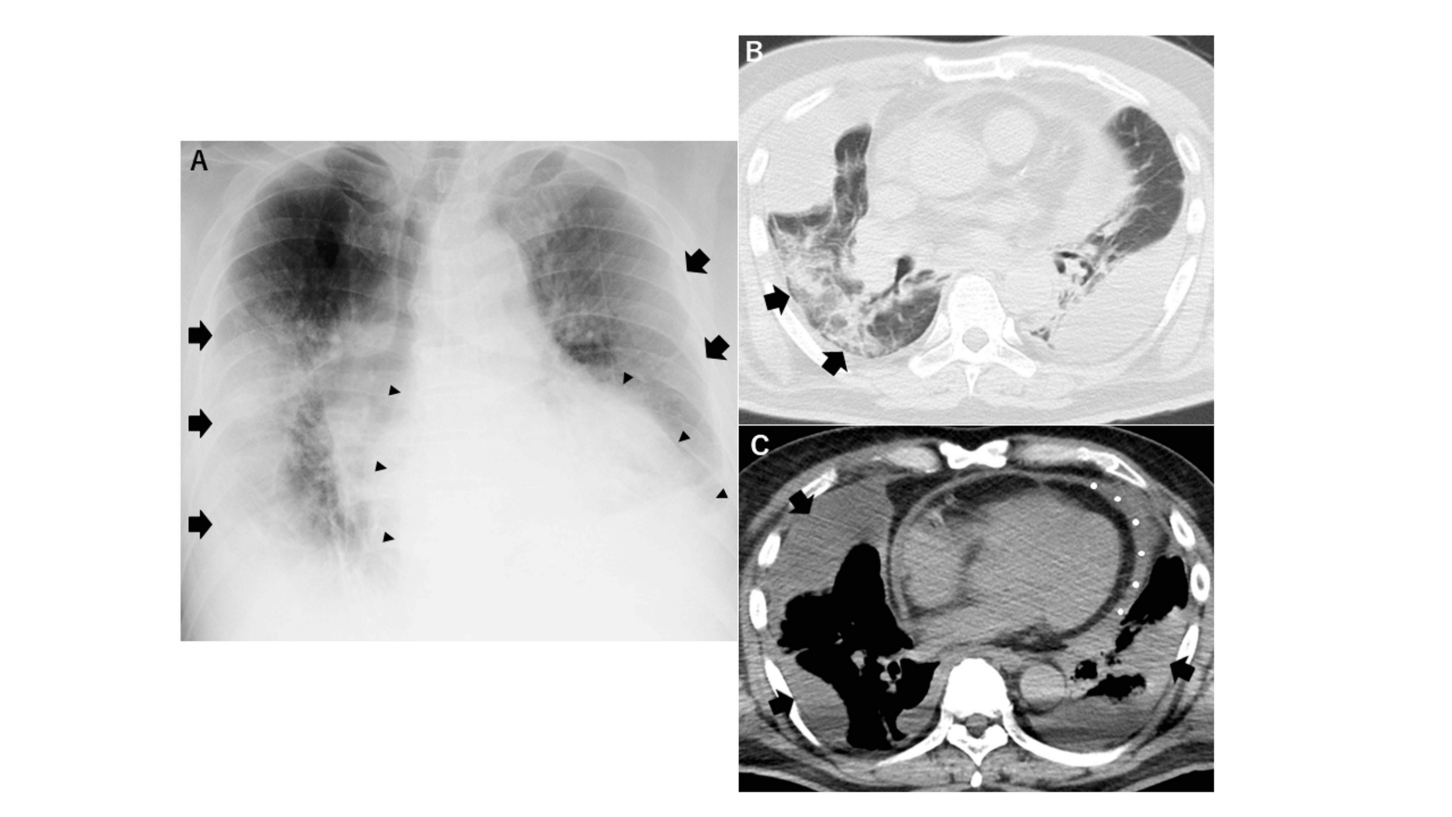
Chest X-ray (A), CT findings of bacterial pneumonia (B), and bilateral empyema and purulent pericardial effusion (C) (A) The chest X-ray shows bilateral pleural infiltration (arrows) and an enlarged cardiac silhouette (triangle marker). (B) There is evidence of infiltration in the lung fields (arrows), suggestive of pneumonia. (C) The X-ray reveals encapsulated pleural effusion (arrows) in both pleural cavities, along with pericardial effusion (white spot).

In addition, the pneumococcal urinary antigen test was positive. A chest X-ray revealed consolidation in the right middle and lower lung fields, along with diffuse ground-glass opacity in the left lung (Figure 1A). CT imaging showed consolidation in the right lung (Figure 1B), suggesting bacterial pneumonia. Furthermore, loculated pleural effusion was observed in both pleural cavities, along with pericardial effusion (Figure 1C). Analysis of the pleural fluid showed a pH of 6.2, glucose of 1.0 mg/dL, and total protein of 4.3 g/dL. The fluid was cloudy and yellow-brown, with a putrid odor. Based on these findings, the diagnosis was empyema.

**Figure 2:**
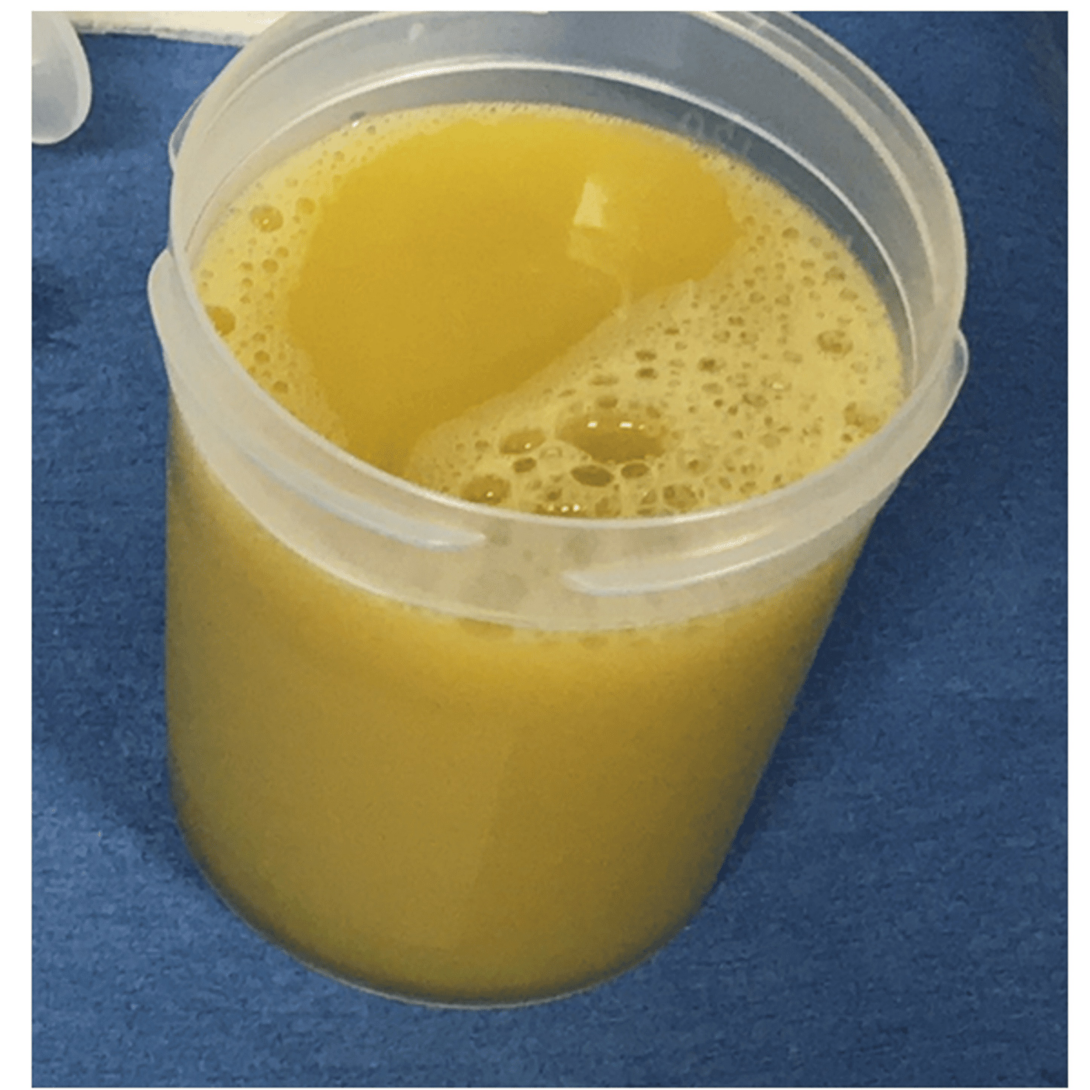
Appearance of the pleural effusion A drain was placed in the bilateral thoracic cavity, and a foul-smelling, cloudy, yellow-brown pleural effusion was drained.

The patient was admitted to the intensive care unit (ICU) for intensive monitoring and management. Due to hypoxemia, tracheal intubation and ventilation were initiated. Ringer's solution, noradrenaline, hydrocortisone, vasopressin, and albumin preparations were administered to manage septic shock. Empiric antibiotic therapy with piperacillin/tazobactam (TAZ/PIPC) and levofloxacin was initiated for pyothorax and pneumonia. Drains were inserted into both thoracic cavities, and malodorous yellow-brown purulent pleural effusions were withdrawn (Figure 2).

Urokinase was then injected into the thoracic cavities to dissolve the intrathoracic capsule, which was then washed with physiological saline. Gram staining of pleural fluid (bilateral), blood cultures (two sets), and sputum all showed Gram-positive cocci, and subsequent cultures confirmed *Streptococcus pneumoniae* after a few days. Antimicrobial susceptibility testing of these specimens showed that it was sensitive to penicillin G (PCG), amoxicillin (AMPC), cefotaxime (CTX), ceftriaxone (CTRX), imipenem/cilastatin (IPM/CS), meropenem (MEPM), quinupristin/dalfopristin (QPR/DRR), rifampicin (RFP), vancomycin (VCM), levofloxacin (LVFX), sulfamethoxazole/trimethoprim (ST), and ofloxacin (OFLX) (Table 2).

**Table 2:** Antimicrobial susceptibility test *Streptococcus pneumoniae *detected in the empyema, blood, and sputum was susceptible to all antibiotics except clindamycin and erythromycin. Based on the results of the susceptibility test, the antibiotic therapy was adjusted. Test performed using Microscan WalkAway 96 Plus system (Beckman Coulter, Brea, California).

Drug susceptibility	MIC (μg/mL)	Susceptibility
Penicillin G	≤0.03	S
Ampicillin	≤0.06	S
Cefotiam	≤0.5	S
Cefotaxime	≤0.12	S
Ceftriaxone	≤0.12	S
Levofloxacin	1	S
Meropenem	≤0.12	S
Erythromycin	>2	R
Clindamycin	0.5	I
Vancomycin	0.5	S

Elevated BUN and creatinine levels suggested a decline in renal function. The rapid deterioration of renal function was thought to be caused by reduced renal blood flow due to hypotension, inflammation, disseminated intravascular coagulation (microthrombus formation), and increased vascular permeability resulting from endothelial damage in sepsis. Continuous hemodiafiltration was initiated due to acute renal failure.

On day 7, cardiac tamponade developed due to an increased pericardial effusion volume. Pericardial drainage was initiated, and yellow-brown purulent pericardial effusion was drained. Drainage continued for one week, but no pneumococci were detected. Although pneumococci were detected in pleural fluid, blood, and sputum samples collected at admission, they were not found in the purulent pericardial fluid obtained on day 7. This was presumed to be due to sterilization of the purulent pericardial fluid by antimicrobial treatment over the past week. However, the causative bacteria of the purulent pericardial fluid were considered to be the same as those responsible for the previous abscess.

**Figure 3:**
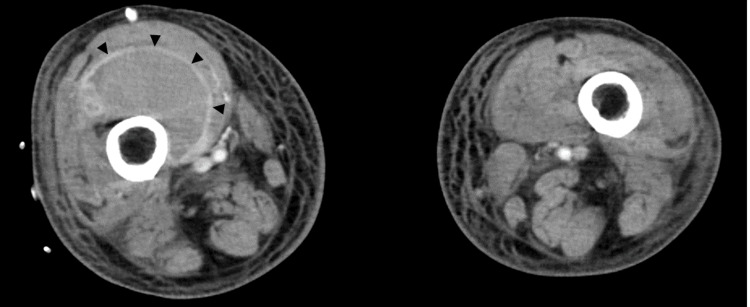
CT findings of an intramuscular abscess The patient presented with pain in the right thigh, and a CT scan revealed an intramuscular abscess in the same region (triangle marker).

Based on the results of the previous antimicrobial susceptibility testing (Table 2), TAZ/PIPC was switched to CTRX. However, the patient’s general condition did not improve, and on day 22, CT revealed an abscess in the right thigh (Figure 3), suggesting a pneumococcal bloodstream infection.

**Figure 4:**
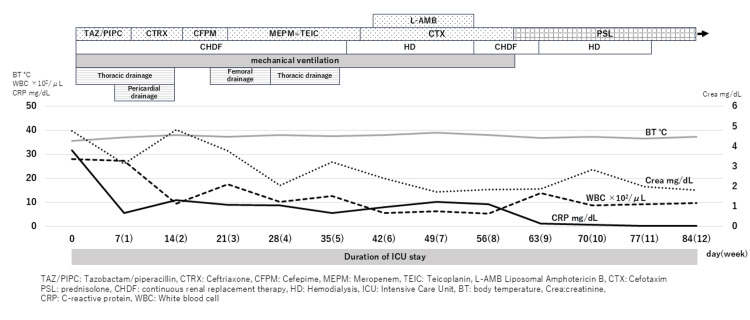
Clinical course during ICU admission Upon hospital admission, the patient had elevated WBC and CRP levels, indicating a state of severe inflammation. He was diagnosed with pneumonia caused by serotype 1 Streptococcus pneumoniae, septic shock, and multiple organ failure, with a particular emphasis on renal failure. He received prolonged treatment with various antimicrobial agents and was administered prednisolone (PSL) for post-infectious organizing pneumonia. He required hemodialysis for 11 weeks and mechanical ventilation for 2 months due to respiratory failure. He developed empyema, bacterial pericardial effusion, and a femoral muscle abscess, all of which required drainage procedures. It took 12 weeks for him to be discharged from the ICU.

Accordingly, a catheter was inserted into the right thigh, and 100 mL of bloody purulent fluid was drained; however, no pneumococci were detected. It was presumed that the thigh abscess, like the purulent pericardial fluid, had been sterilized by prior antimicrobial treatment. Antibiotic therapy was then changed again from CTRX to cefepime, TAZ/PIPC, and MEPM + teicoplanin. After 39 days, Candida guilliermondii was detected in the patient’s blood, and L-AMB was administered for two weeks. On day 67, prednisone 80 mg (1 mg/kg/day) was started for post-infectious organizing pneumonia. At this time, the patient’s general condition began to improve; renal function recovered, and dialysis was discontinued by day 79. After respiratory status improved, the patient was weaned from the ventilator on day 80 and discharged from the ICU on day 84 (Figure 4). The tracheostomy was closed on day 238. However, the patient was still unable to walk or use a wheelchair independently and required assistance with daily activities. His Activities of Daily Living (ADL) and general condition had not returned to pre-onset levels, and he was transferred to another hospital for further intensive rehabilitation.

*Streptococcus pneumoniae* isolated from the empyema fluid was sent to the National Institute of Infectious Diseases for further analysis, which identified the strain as serotype 1.

## Discussion

This study reported the case of a 68-year-old unvaccinated man who developed IPD with multiple systemic abscesses in various sites from serotype 1 pneumococcus and was hospitalized for 10 months, emphasizing the importance of vaccination for the elderly and immunocompromised individuals.

According to a systematic review of 26 papers from multiple countries published by Chen et al., the mortality rate of IPD in Japan is 20% [7]. As the condition is particularly high in children and the elderly [8], IPD prevention is a major issue in this country.

In Japan, the 7-valent pneumococcal conjugate vaccine (PCV7: against serotypes 4, 6B, 9V, 14, 18C, 19F, and 23F) was voluntarily introduced for children in 2010, officially incorporated into the vaccination program for public administration, and replaced with PCV13 (PCV7 + serotypes 1, 3, 5, 6A, 7F, and 19A) in 2013. After that introduction, the number of pediatric patients with IPD decreased from 300 in 2010 to 156 in 2012, with PCV7 serotype IPD decreasing from 73.3% in 2010 to 14.7% in 2012 in children, and non-PCV7 serotype IPD increasing from 26.7% in 2010 to 85.3% in 2012 [9]. This suggests that the overall proportion of vaccine-type IPD decreased, whereas the proportion of non-vaccine-type IPD increased. The number of cases of serotype 1 IPD increased from 0 in 2010 to 5 (1.4%) in 2011-2013 and 13 (3.7%) in 2014-2016 (p < 0.001). Serotype 1 is a non-PCV7 type, and the incidence of serotype 1 IPD increased after the introduction of PCV7. PCV13 has been inoculated since 2013, covering serotype 1, and the number of serotype 1 IPD cases has been decreasing since that time [10].

Similarly, in adults, PCV7-type IPD decreased, and non-PCV7-type IPD increased after 2010. Additionally, since the introduction of PCV13 in 2013, the incidence of PCV13 serotype IPD has decreased, indicating that childhood vaccination influences IPD serotypes in adulthood [10,11]. PCV7 does not contain serotype 1, which may have increased the number of children colonized with serotype 1 pneumococci after the introduction of PCV7. The patient spent several days with a school-aged granddaughter who had rhinovirus symptoms, and it is possible that he contracted pneumococcus from her.

IPD due to serotype 1 is rare: of the 715 specimens collected in Japan from April 2010 to March 2013, only two tested positive for serotype 1 IPD [10]. The risk of death varies depending on the serotype, and the mortality rate for IPD caused by serotype 1 is said to be low. In their study, Weinberger et al. showed that pneumonia and meningitis caused by serotypes 3, 6A, 6B, 9N, and 19F had a high risk of death, while serotypes 1, 7F, and 8 had a low risk of death [12]. Similar results were found in the study by van Hoek et al., in which serotypes 19F (41%), 31 (40%), and 3 (39%) had high mortality rates, whereas serotypes 1 (17%), 7F (20%), and 12F (21%) had lower mortality rates [13]. In addition, van Hoek et al. reported that serotype 1 is more likely to develop empyema [13]. The infection focus differs depending on the serotype. Serotypes 35F (13%), 6C (10%), and 18C (10%) were more likely to cause meningitis in a population. Serotype 1 (6%) was the most prevalent for empyema, followed by 7F and 12F (both 3%) in individuals aged 65 years and older [13]. The case of IPD caused by serotype 1 that we encountered was a serious case that did not lead to death but caused multiple abscesses and required long-term ICU management.

Host IPD risk factors include age, chronic heart disease, chronic lung disease, diabetes, functional or anatomical asplenia, chronic liver disease, cancer-bearing status, autoimmune disease, immunosuppression, smoking, and alcohol use [14-19]. In this case, old age and diabetes were risk factors; however, the patient’s HbA1c was 6.8, showing well-controlled diabetes, suggesting that age was the only substantial risk factor.

## Conclusions

In conclusion, serotype 1 is less likely to cause IPD and has a low fatality rate. In this case, the only identified risk factor for IPD was advanced age; however, the patient developed a severe form of the disease. He developed empyema, purulent pericardial effusion, and a thigh abscess, which led to sepsis, multiple organ failure, a prolonged ICU stay, extended rehabilitation, and a significant decline in ADL and quality of life. Since serotype 1 is included in both PPSV23 and PCV13, vaccination with either could have potentially prevented this severe illness. This case highlights the importance of pneumococcal vaccination for individuals over 65 years of age, even in the absence of traditional risk factors.

In addition, childhood vaccination influences the circulating serotypes of IPD. Due to herd immunity, these changes also affect the serotype distribution of IPD in adults. In the near future, there may be an increase in serotype 1 IPD among adults, making it necessary to continue monitoring these trends.
